# Stylostome formation by parasitic larvae of *Allothrombium fuliginosum* (Trombidiformes: Trombidiidae): morphology of feeding tubes and factors affecting their size

**DOI:** 10.1007/s10493-020-00553-8

**Published:** 2020-09-29

**Authors:** Magdalena Felska, Joanna Mąkol, Andrey B. Shatrov

**Affiliations:** 1 Institute of Biology, Department of Invertebrate Systematics and Ecology, Wrocław University of Environmental and Life Sciences, Kożuchowska 5b, 51-631 Wrocław, Poland; 2 Zoological Institute of the Russian Academy of Sciences, St. Petersburg, Russia 199034

**Keywords:** Ectoparasites, Feeding tubes, Parasitengona, Host–parasite interaction, Ultrastructure

## Abstract

The morphology and formation of stylostomes (feeding tubes) in hosts’ body during the parasitic phase of *Allothrombium fuliginosum* (Hermann) larvae were studied for the first time with light microscopy (LM) and transmission electron microscopy (TEM). The stylostomes were observed in three aphids species—*Acyrthosiphon pisum* (Harris), *Elatobium abietinum* (Walker), and *Macrosiphum rosae* (L.)—parasitized by mites under laboratory conditions. They consisted of 2–6 main branches, preliminarily unbranched, then producing secondary and sometimes also tertiary branches as finally formed structures. Their walls were uniformly electron-dense, without any longitudinal and transverse stratifications and showed rather irregular outlines. Distally, the stylostome branches revealed transparent pores and cavities in their walls, connecting the stylostome canal with surrounding haemocoelic space. The total length of stylostomes at the end of the parasitic phase was on average 16× greater than that recorded in the youngest stylostomes. No differences in the overall shape of feeding tubes between host species were stated. The stylostomes formed in different host species did not differ significantly, except their total length, which attained the highest value in tissues of *Ac. pisum*.

## Introduction

Parasitism by larvae of some Parasitengona mites is associated with the formation of stylostomes (feeding tubes) in the hosts’ tissues. Larvae feed on the host's haemolymph and liquefied host material (Peterson et al. 1992; Wohltmann 2000; Shatrov 2009; Shatrov et al. 2014). There has been much debate as to whether the stylostomes are organs of the mites, salivary secretions of the mite, a reaction by the host, or a combination of the latter two, but the current consensus is that they result from salivary secretions (Davids 1973; Åbro 1979; Redmond and Hochberg 1981; Wohltmann 2000; Smith 2003; Shatrov and Felska 2017). The first references to these structures go back to the nineteenth century (Gudden 1871; Flögel 1876; Jourdain 1892, 1899; Trouessart 1897, 1899). Until present, stylostomes have been reported for several Hydrachnidia, Trombiculidae, and Trombidiidae (*Trombidium*, *Allothrombium*) mites (Wohltmann 2000). Feeding tubes of these taxa differ significantly. Trombiculid larvae, being parasites of vertebrates, produce relatively wide, always un-branched, and open-ended stylostomes (Voigt 1970; Wohltmann 2000; Shatrov 2009; Shatrov et al. 2014). Most early-derivative water mite superfamilies have multiply-branched stylostomes (Eylaoidea, Hydrachnoidea, Hydryphantoidea), whereas later-derived ones (Hygrobatoidea, Arrenuroidea) have unbranched, closed-ended stylostomes (Smith 2003). Stylostomes of Trombidiidae have been considered multibranched and blind-ended (Wohltmann 2000).

In Trombidiidae, the stylostomes have been described to date for members of *Trombidium*: *Trombidium holosericeum* (L.) (Jourdain 1899; Mąkol and Wohltmann 2000; Shatrov and Felska 2017), *Trombidium brevimanum* (Berlese) (Wohltmann 1999; Judson and Mąkol 2011), *Trombidium newelli* Welbourn et Flessel, in Peterson et al. (1992) (*nomen nudum*, see Mąkol and Wohltmann 2013) (Mohamed and Hogg 2004), *Trombidium mediterraneum* (Berlese) (Judson and Mąkol 2011), *Trombidium* sp. (Wharton 1954) and *Trombidium susteri* (Feider) (*nomen dubium*, see Mąkol 2005) and for only one representative of *Allothrombium*—*Allothrombium recki* Feider et Agekian (Feider and Agekian 1967; Robaux 1974).

Members of *Allothrombium* spp., due to their potential for biological control of insect and mite pests, have long attracted the attention of researchers (Zhang and Xin 1989a, 1989b; Zhang 1991a, 1991b, 1992, 1998; Zhang and Chen 1993; Zhang et al. 1993). Despite attempts to determine the overall effect of parasitic larvae and predatory post-larval forms on the host/prey population, detailed knowledge of the interaction remains scanty. *Allothrombium fuliginosum*, being one of the most common members of the genus, is widely distributed in the Palaearctic (Felska et al. 2018; Mąkol et al. 2019); its larvae parasitize mainly Aphididae (Hemiptera), but were also reported from Araneae, Lepidoptera, Orthoptera and Hymenoptera (Felska et al. 2018).

The present study aims to provide a detailed description of stylostomes produced by *A. fuliginosum* larvae, using light microscopy (LM) and transmission electron microscopy (TEM) methods. The observations are intended to show how the stylostome develops during larval parasitism and to answer the question, whether factors such as host species and host size influence stylostome morphology.

## Material and methods

Active postlarval forms of *A. fuliginosum* were captured by hand by the senior author in March and April 2019 (51° 09′ 83.93″ N, 17° 09′ 42.28″ E, at the campus of the Wrocław University of Environmental and Life Sciences, Wrocław, Poland). The specimens were transferred into separate glass rearing vials (34 × 24 mm) filled with charcoaled plaster-of-Paris and covered with tight, semi-transparent plastic lids. At all stages of the experiment, the mites were kept in environmental test chamber Sanyo MLR-351H (Sanyo Electric, Osaka, Japan), at fixed humidity (80% RH), temperature (22 °C day/15 °C night), and photoperiod (12 h/12 h L:D). The content of the vials was checked at regular 3-day intervals in order to record the oviposition events and larval emergence.

Larvae obtained from field-collected, ovigerous females were intended for parasitism experiments within the first 3 days after emergence. For purpose of stylostome studies three aphid species were offered as hosts: the pea aphid, *Acyrthosiphon pisum* (Harris), the green spruce aphid, *Elatobium abietinum* (Walker), and the rose aphid, *Macrosiphum rosae* (L.). *Acyrthosiphon pisum* aphids were kept on pea sprouts grown in planter pots. *Elatobium abietinum* and *M. rosae* were collected by the senior author, together with branches of host plants, *Picea pungens* and *Rosa* × *damascena*, respectively, for each aphid species, in a private garden (51° 16′ 24.85″ N, 17° 25′ 62.81″ E, Bielawa ad. Wrocław, Poland) in May and June 2019. The material was kept in the environmental test chamber under the conditions specified above. The experiments were set in 2-L glass beakers (250 × 135 mm), separately for each host species. Pieces of the host plant with aphids were introduced to the bottom of the beaker (one pot with pea bush or spruce/rose twigs placed on moist lignin), followed by the addition of several dozen of freshly hatched larvae. The top of each glass beaker was tightly covered with screen printing mesh (80 µm mesh) and elastic band.

For comparison of stylostomes produced in tissues of different host species, aphids were used with larvae between the second and third day of parasitism (24–72 h). In order to trace stylostome formation in successive time intervals, the tissues of *Ac. pisum* were examined. Hosts with larvae were transferred with soft tweezers to a fixative (2.5% glutaraldehyde for TEM studies, 80% lactic acid for LM study) every 2 h during the first 12 h of the parasitic phase and once a day in the following days (i.e., 2–12, 24, 48, 72 h, etc.), until termination of the parasitic phase. The material was kept in lactic acid, then heated to 70 °C for 15 min in a heating block and fixed on microscope slides in Hoyer’s medium prior to LM examination.

Photos of *A. fuliginosum* larvae parasitizing *Ac. pisum* were taken using a Nikon SMZ800 stereomicroscope coupled with a Toshiba 1080i camera system. Stylostome measurements (given in micrometres) were taken under a Nikon Eclipse E600 compound light microscope coupled with differential interference contrast (DIC) and NIS Elements BR software. For morphometric analyses, only fully visible and clear stylostomes were used. Measurements included: number and total length of main branches (measured along a curved line between stylostome base and its most distal part), number of secondary branches departing from the main canal and the sum of their length, number of tertiary branches departing from the secondary branches and the sum of their length, total length of the stylostome (total length of all branches), diameter of the canal lumen (measured in the most basal part), diameter of the main branch (measured in the most basal part, at the mid-point, and in the most distal part). Host length was measured from the base of antennae to the end of abdomen, host width was measured at the level of the third pair of legs.

Statistical analyses were performed in STATISTICA v.13.3 (Tibco Software 2017). A Kruskal–Wallis test was used to compare stylostomes produced among host species. To check the correlation between the length of the parasitic phase of *A. fuliginosum* on pea aphids and the size of the stylostomes as well as the hosts’ body size, Spearman's rank correlation coefficient was used.

For transmission electron microscopy (TEM), standard double fixation in 2.5% glutaraldehyde in 0.1 M cacodylate buffer (pH 7.2–7.4) and 2% osmium tetroxide in 0.1 M cacodylate buffer was applied for the whole aphids (*Ac. pisum*) with feeding larvae (see Shatrov and Felska 2017). The samples were then dehydrated in ethanol and acetone series and finally embedded in an araldite mixture (Fluka). Serial ultra-thin sections, in perpendicular planes to the integument of the host body, were made using Leica UC-6 ultramicrotome (Leica, Wetzlar, Germany) and mounted on copper grids with an oval hole provided with formvar support. Sections were stained with uranyl acetate and lead citrate (Reynolds 1963) and examined and photographed with a Morgagni 268-D (FEI Company, Hillsboro, OR, USA) transmission electron microscope at 80 kV (digital visualization). For preliminary and general observations, semi-thin sections from the same blocks were stained with toluidine blue and examined with the Leica DM LS-2 light microscope coupled with Leica EC-3 digital camera system. In total, five samples of the host body portions with larvae at approximately medium stage of feeding were studied in TEM.

For additional scanning electron microscopy (SEM) of the mouth apparatus, unfed alcohol-preserved larvae of *A. fuliginosum* were rinsed in graded ethanol series and cleaned in an ultrasonic cleaner for 3–4 min. Larvae were then dried at critical point of carbonic acid in a Hitachi HCP-2 vacuum evaporator, or rinsed in 96% ethyl alcohol for 10–15 min and then treated with hexamethyldisilazane (HMDS) for 5–10 min as an alternative to critical point drying. Larvae were then covered with a platinum layer in the Eiko IB-5 apparatus and examined with a Quanta 250 (FEI Company) scanning electron microscope at 10–15 kV.

## Results

### Scanning electron microscopy (SEM)

**Fig. 1:**
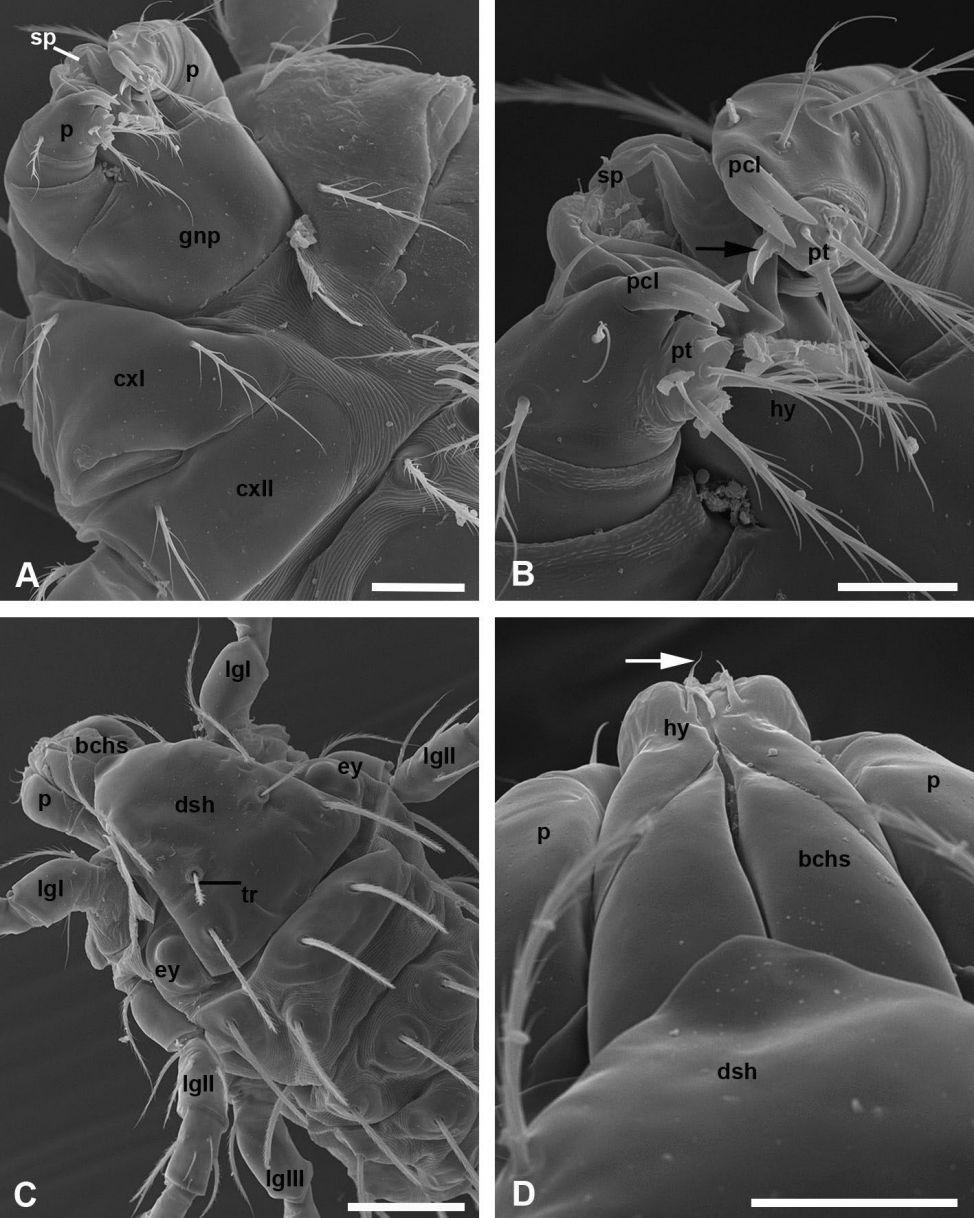
**a**–**d** Mouth apparatus of *Allothrombium fuliginosum* larvae. SEM micrographs. **a** Ventral view of the anterior body portion. Scale bar: 25 µm. **b** Ventral view of gnathosoma in higher magnification. Note bifurcate palpal claws and a flexible apical portion of the hypostome forming a suction pad functioning as a temporary sucker. Also note the hypertrophied spoon-like solenidion in apical part of the palp tarsus (*arrow*). Scale bar: 10 µm. **c** Dorso-lateral view of the frontal body portion. Scale bar: 50 µm. **d** Dorsal view of the mouth apparatus. Note small papillae (setae *cs*) extending from the hypostomal portions enveloping the chelicerae (*arrow*). Scale bar: 20 µm. *bchs* basal cheliceral segment, *cxI* coxa of leg I, *cxII* coxa of leg II, *dsh* dorsal shield (scutum), *ey* eye, *gnp* gnathocoxal plate, *hy* hypostome, *lgI–lgIII* legs I–III, *p palp*, *pcl* palpal claw (odontus), *pt* palp tarsus, *sp* suction pad, *tr* trichobothrium

SEM of the mouth apparatus of unfed larvae reveals the possibility of formation of the temporary sucker (Fig. 1a). It may be formed of the flexible distal hypostome portion, which does not bear any papillae and lamellae on the sucker as such (Fig. 1b). Only one pair of small papillae extending straight forward and known as ‘*cs*’ setae in taxonomic descriptions, is located in antero-dorsal position (Fig. 1d). During feeding, the suction pad attaches to the host cuticle, apparently facilitating the action of the pharyngeal pump (Figs. 4c, 5a, d). In the non-feeding condition, the cheliceral movable digits are retracted and cannot be seen externally. The bifurcate palpal claws (Fig. 1b) do not penetrate the host integument. Each palp tarsus is provided with one distinctly flattened solenidion having a slightly concave ventral surface and oriented medially (Fig. 1b). Both the palpal claws and the solenidia are covered with a fine cuticular striation.

From above, the basal portion of the cheliceral base is covered with an anterior protrusion of the scutum (frontal dorsal shield) (Fig. 1c, d).

### Light microscopy

**Fig. 2:**
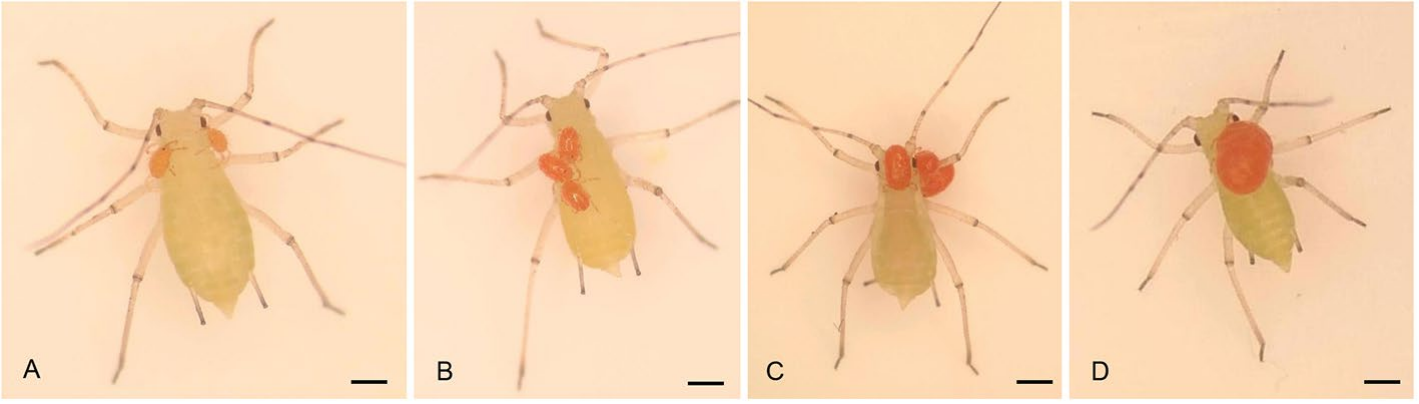
**a**–**d** Larvae of *Allothrombium fuliginosum* at different degree of engorgement on *Acyrthosiphon pisum*. **a** 2 h after attachment to the host; **b** 24 h; **c** 72 h; **d** 144 h. Scale bars: **a**–**c** 450 µm; **d** 250 µm

Parasitism was observed already a few minutes after introducing the larvae to the containers with aphids. In the case of *Ac. pisum* the parasitic phase lasted 6 days on average (5–8, *n* = 50) and a gradual increase in body size of the larva could be observed (Fig. 2a–d). After transfer to the preservative, most parasitic larvae immediately detached themselves from the host.

**Fig. 3:**
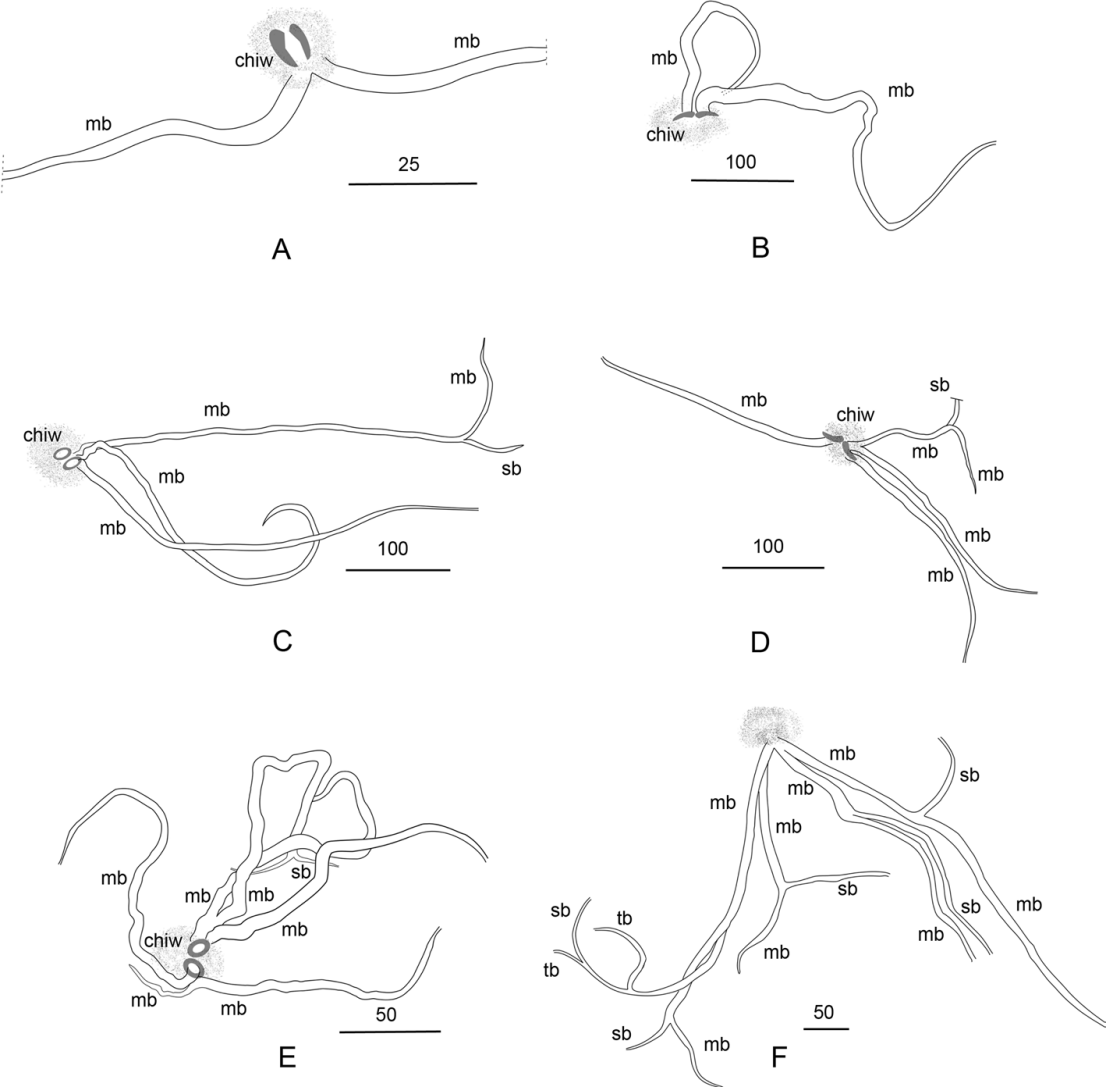
**a**–**f** Stylostome of *Allothrombium fuliginosum* in tissues of aphid *Acyrthosiphon pisum*. **a** 10 h after attachment to the host; **b** 24 h; **c**, **d** 48 h; **e** 96 h; **f** 168 h. *chiw* internal wound left by mite chelicerae, *mb* main branch, *sb* secondary branch, *tb* tertiary branch

**Fig. 4:**
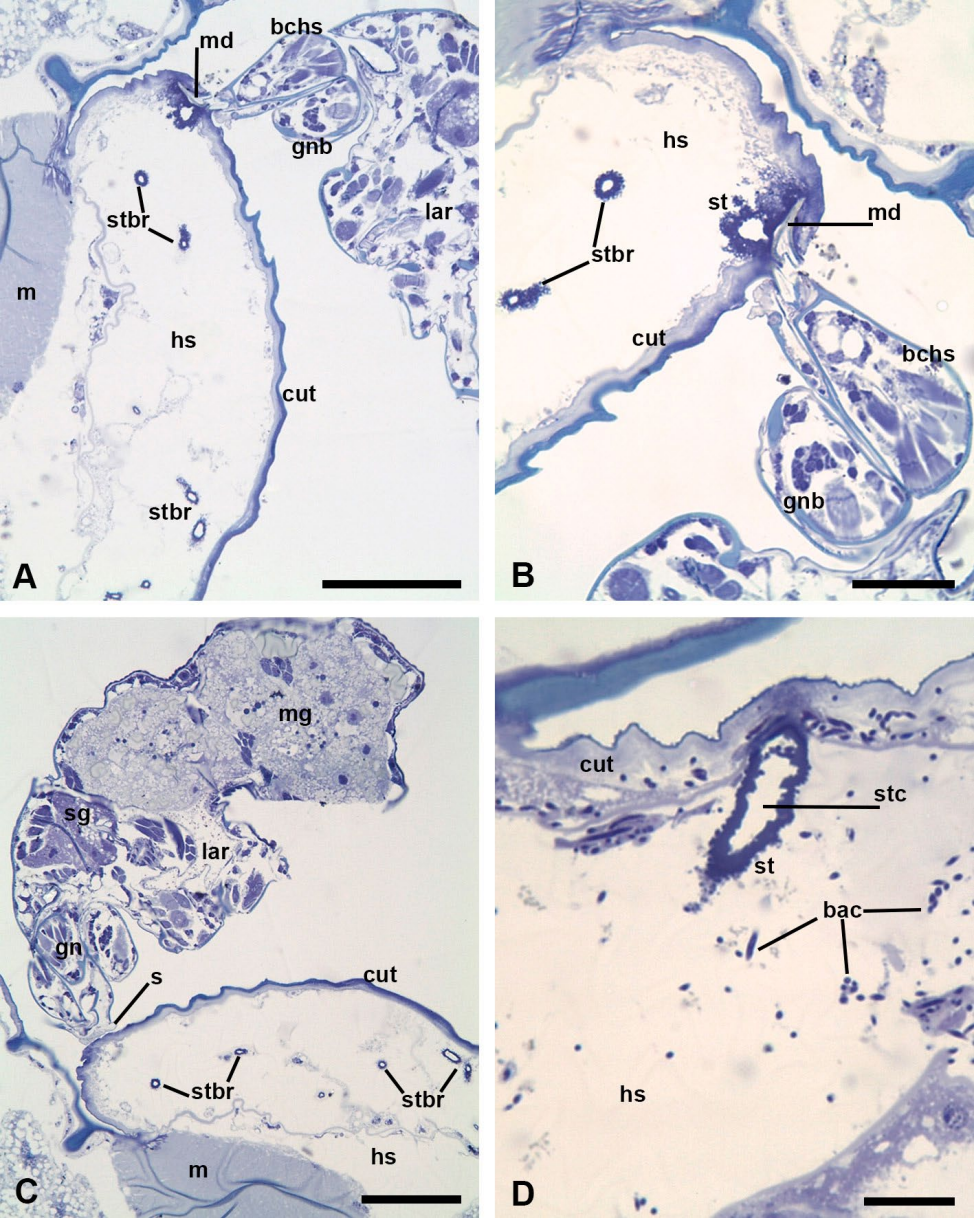
**a**–**d** Semi-thin toluidine blue stained sections of aphids (*Acyrthosiphon pisum*) parasitized by larvae of *Allothrombium fuliginosum*. **a** Lateral body portion of an aphid in cross section with an attached larva. Scale bar, 50 µm. **b** Mouth apparatus of an attached larva showing protruded cheliceral movable digits (cheliceral blades) cutting the host cuticle and a proximal portion of the stylostome. Note stylostome branches on nearly cross sections. Scale bar, 20 µm. **c** Para-sagittal section of larvae attached to the host cuticle. Note sucker tightly adjoined to the cuticle and numerous stylostome branches. Scale bar, 50 µm. **d** Oblique section through the main branch of the stylostome. Note bacteria in the zone of affection. Scale bar, 10 µm. *bac* bacteria, *bchs* basal cheliceral segment, *cut* host cuticle, *gn* gnathostoma, *gnb* gnathobase, *hs* hemacoelic space, *lar* larva, *m* host muscles, *md* cheliceral movable digits (cheliceral blades), *mg* midgut, *s* sucker, *sg* salivary glands, *st* stylostome, *stbr* stylostome branches, *stc* stylostome canal

The feeding tubes, starting at the larval gnathosoma, spread throughout the tissues of the insect. The ramified stylostomes consist of 2–6 main branches, which diverge into different directions (Fig. 3a–f). These main stylostome tubes are either unbranched (Figs. 3a, b, 4b) or produce secondary branches (Fig. 3c–f), which are shorter and thinner than the main branches and develop mostly in the distal parts of the stylostome (up to seven such branchings have been observed within the entire structure). In some stylostomes (four out of 48 analyzed), relatively short, tertiary branches are observed (Fig. 3f), much smaller in diameter than the superior branches. The most proximal part of the stylostome is either unbranched or several independent branches are formed already at the stylostome base. On the 2nd and 3rd days from the onset of parasitism, the stylostomes usually consist of three main branches. The most distally located tubules narrow gradually until they are undetectable in the host tissues. The walls of all stylostome canals have convexities and concavities (the character visible using 40× and 100× objective lenses). In case several stylostomes are formed in the tissues of the same host specimen, their canals always remain isolated from each other. The metric data on feeding canals recorded in the tissues of three different host species are summarized in Table 1.

**Table 1:** Mean (min–max) of morphometric data on stylostomes produced by *Allothrombium fuliginosum* in three host species and basic measurements of the hosts

Variable	*Acyrthosiphon pisum*	*Elatobium abietinum*	*Macrosiphum rosae*	Kruskal–Wallis H	p
Sample size (stylostomes/aphids)	10/9	10/8	10/8	–	–
Number of main branches	3.3 (2–6)	3 (2–4)	3.1 (2–5)	0.285	0.87
Total length of main branches (µm)	956 (326–1549)	564 (156–1120)	514 (297–907)	5.667	0.059
Number of secondary branches	1.1 (0–4)	0.5 (0–2)	1 (0–3)	1.266	0.53
Total length of secondary branches (µm)	150 (72–497), n = 5^a^	254 (119–524), n = 3^a^	108 (23–249), n = 5^a^	0.192	0.91
Total length of all branches (µm)	1038 (419–1621)	640 (156–1349)	568 (297–907)	6.08	0.048
Diameter of main branch at base (µm)	8 (5.6–11.8)	8.1 (5.7–11.3)	7.8 (5.6–9.5)	0.217	0.90
Diameter of the canal lumen at base (µm)	4 (2.8–5.9)	5.5 (2–7.5)	3.7 (2.8–5)	2.77	0.25
Diameter of main branch at mid-point (µm)	6.8 (4.6–9.6)	6.4 (4.7–7.9)	6.3 (4.2–7.4)	0.528	0.77
Diameter of main branch at termination (µm)	2.9 (2.3–3.6)	2.5 (1.4–3.6)	2.7 (2.1–4.1)	3.268	0.20
Host body length (µm)	2188 (1775–2600)	1622 (1348–1971)	2582 (2022–3661)	–	–
Host body width (µm)	971 (812–1233)	867 (632–1191)	1177 (864–1620)	–	–

^a^Number of stylostomes with secondary branches and without tertiary branches observed

The youngest observed stylostome was visible in the pea aphid (*Ac. pisum*) tissues 10 h from the onset of larval parasitism. The 1-day-old stylostomes consisted exclusively of main canals and were devoid of secondary branchings. The mean total length of the stylostomes produced in tissues of *Ac. pisum* by the larvae completing the parasitic phase (on the 7th day) was on average 16× higher compared to the youngest observed stylostome. The external diameter and the diameter of the lumen at the base of the main branches and at the mid-point of mature stylostomes were on average 3× higher compared to the youngest observed structure. The morphometric data on stylostomes produced in tissues of *Ac. pisum* in subsequent steps of the parasitic phase are provided in Table 2.

**Table 2:** Mean (min–max) of measurements of *Allothrombium fuliginosum* stylostomes produced in tissues of *Acyrthosiphon pisum* in subsequent steps of the parasitic phase

Variable	Duration of parasitism (h)							
	10	24	48	72	96	120	144	168
Sample size (stylostomes/aphids)	1/1	3/3	9/8	5/4	3/3	2/2	3/3	2/2
Number of main branches	2	3 (2–4)	3 (2–6)	3.2 (2–4)	3.3 (3–4)	4 (3–5)	3.7 (3–4)	3 (2–4)
Total length of main branches (µm)	120	724 (455–1239)	892 (326–1485)	1270 (695–1686)	665 (625–709)	1094 (455–1734)	1134 (557–2133)	1367 (1142–1593)
Number of secondary branches	0	0	1.2 (0–4)	2 (0–4)	4 (2–6)	5 (3–7)	4	4.5 (3–6)
Total length of secondary branches (µm)	–	–	219 (72–497); n = 5^a^	253 (72–408); n = 4^a^	248 (193–334); n = 3^a^	201 (188–214); n = 2^a^	358 (275–437); n = 3^a^	584 (524–643); n = 2^a^
Number of tertiary branches	0	0	1	0	1	0	1	2^b^
Total length of tertiary branches (µm)	–	–	16	–	15	–	18	142^b^
Total length of all branches (µm)	120	724 (455–1239)	1014 (419–1485)	1472 (968–2094)	918 (854–959)	1295 (669–1922)	1498 (918–2570)	2022 (1666–2378)
Diameter of main branch at base (µm)	4.2	7.1 (5.4–8.7)	7.7 (5.6–11.8)	8.4 (7.1–9.9)	10.3 (10–10.9)	6.4 (5.7–7)	11.4 (9.9–13.7)	9.8 (8.7–11)
Diameter of the canal lumen at base (µm)	2	3.3 (2.7–3.7)	3.9 (2.8–5.9)	4.4 (3.8–5.6)	5 (4.7–5.5)	3.6 (3.2–3.9)	6.6 (4.3–8.5)	5.6 (5.2–6)
Diameter of main branch at mid-point (µm)	3.8	4.8 (3.7–5.8)	6.6 (4.6–9.6)	7.6 (6.4–8.8)	7.5 (7–8.3)	6.7 (5–8.3)	8.3 (7–10)	11.8 (9–14.7)
Diameter of main branch at termination (µm)	2.6	2.2 (1.7–2.6)	2.9 (2.3–3.6)	3.1 (2.7–3.6)	3 (2.8–3.3)	3 (1.8–4.1)	3.1 (2.6–3.8)	3.2 (2.3–4.1)
Host body length (µm)	708	875 (665–1043)	927 (812–1111)	1093 (780–1233)	816 (658–1095)	770 (640–901)	911 (803–1024)	1055 (908–1201)
Host body width (µm)	1445	1885 (1265–2225)	2090 (1775–2600)	2402 (1873–2838)	1890 (1366–2609)	1792 (1646–1939)	2042 (1772–2432)	2399 (1973–2825)

^a^Number of stylostomes with secondary branches

^b^Two tertiary branches observed within one stylostome

### Statistical analyses

Differences in the stylostome morphometric data among host species were not significant, except the total length of the feeding tubes, which attained the highest value in tissues of *Ac. pisum* (Table 1). Spearman's rank correlation coefficient showed a positive and significant relationship between duration of the parasitic phase and total length of the stylostome, number and total length of secondary branches, and diameter of main canals, except for diameter of the main branches in the most distal part (Table 3). The overall length of the stylostome was strongly positively correlated with the length of main branches, but it was also correlated with the length of secondary branches, the diameter of the main canal at half of the stylostome length, and the duration of parasitism (Table 3). No correlation between the overall length of the stylostome and the length and width of the host body was found.

**Table 3:** Spearman’s rank correlation coefficients between the duration of parasitism of *Allothrombium fuliginosum* larvae on *Acyrthosiphon pisum* and the total length of the stylostome and other variables

Variable	Correlation coefficients	
	Parasitism duration	Stylostome total length
Parasitism duration	–	**0.49**
Number of main branches	0.32	0.16
Total length of main branches	0.3	**0.92**
Number of secondary branches	**0.77**	0.36
Total length of secondary branches	**0.72**	**0.46**
Number of tertiary branches	0.31	0.17
Total length of tertiary branches	0.32	0.17
Stylostome total length	**0.49**	–
Diameter of main branch at base	**0.52**	0.03
Diameter of the canal lumen at base	**0.65**	0.34
Diameter of main branch at mid-point	**0.65**	**0.42**
Diameter of main branch at termination	0.31	0.33
Host body length	0.07	0.31
Host body width	0.08	0.17

*Bold indicates statistical significance (p < 0.05)

### Transmission electron microscopy (TEM)

**Fig. 5:**
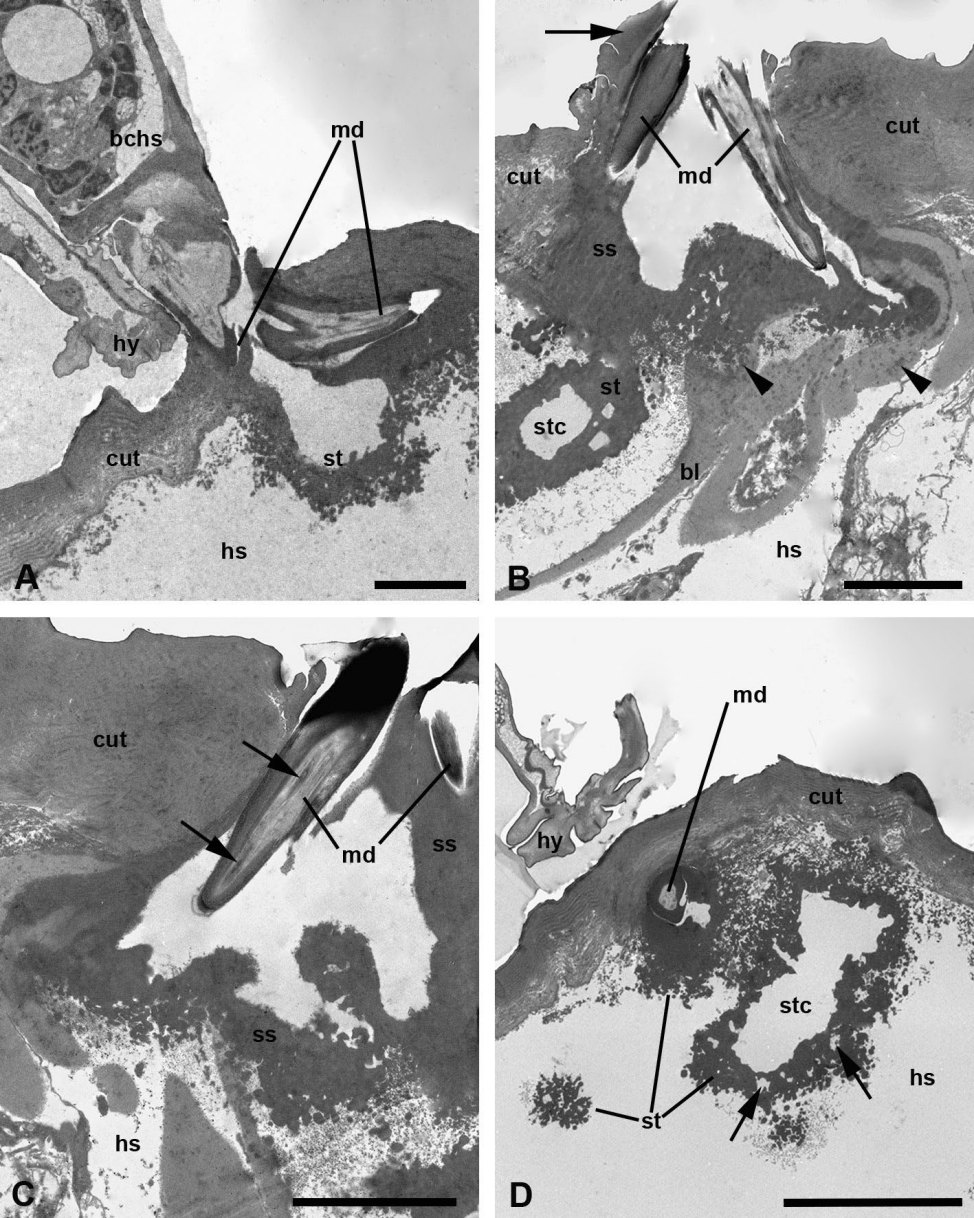
**a**–**d** Stylostome of *Allothrombium fuliginosum* larvae. TEM micrographs. **a** Proximal stylostome portion underneath the cheliceral movable digits piercing the host cuticle and moved apart. Scale bar, 5 µm. **b** Proximal stylostome portion and the cheliceral movable digits penetrating the host integument. Note the stylostome substance protruding above the host cuticle (*arrow*) and the stylostome substance penetrating the basal lamina (*arrowheads*). Scale bar, 5 µm. **c** Cheliceral movable digits cutting the host cuticle and a lucent camera underneath within the proximal stylostome portion. Note dendrites running along the digit (*arrows*). Scale bar, 5 µm. **d** Expanded proximal stylostome portion underneath the cuticle pierced by the cheliceral movable digits. Note lacunas in the stylostome wall (*arrows*). Scale bar, 10 µm. *bchs* basal cheliceral segment, *bl* basal lamina, *cut* cuticle, *hs* haemocoelic space, *hy* hypostome, *md* movable digit, *ss* stylostome substance, *st* stylostome, *stc* stylostome canal

**Fig. 6:**
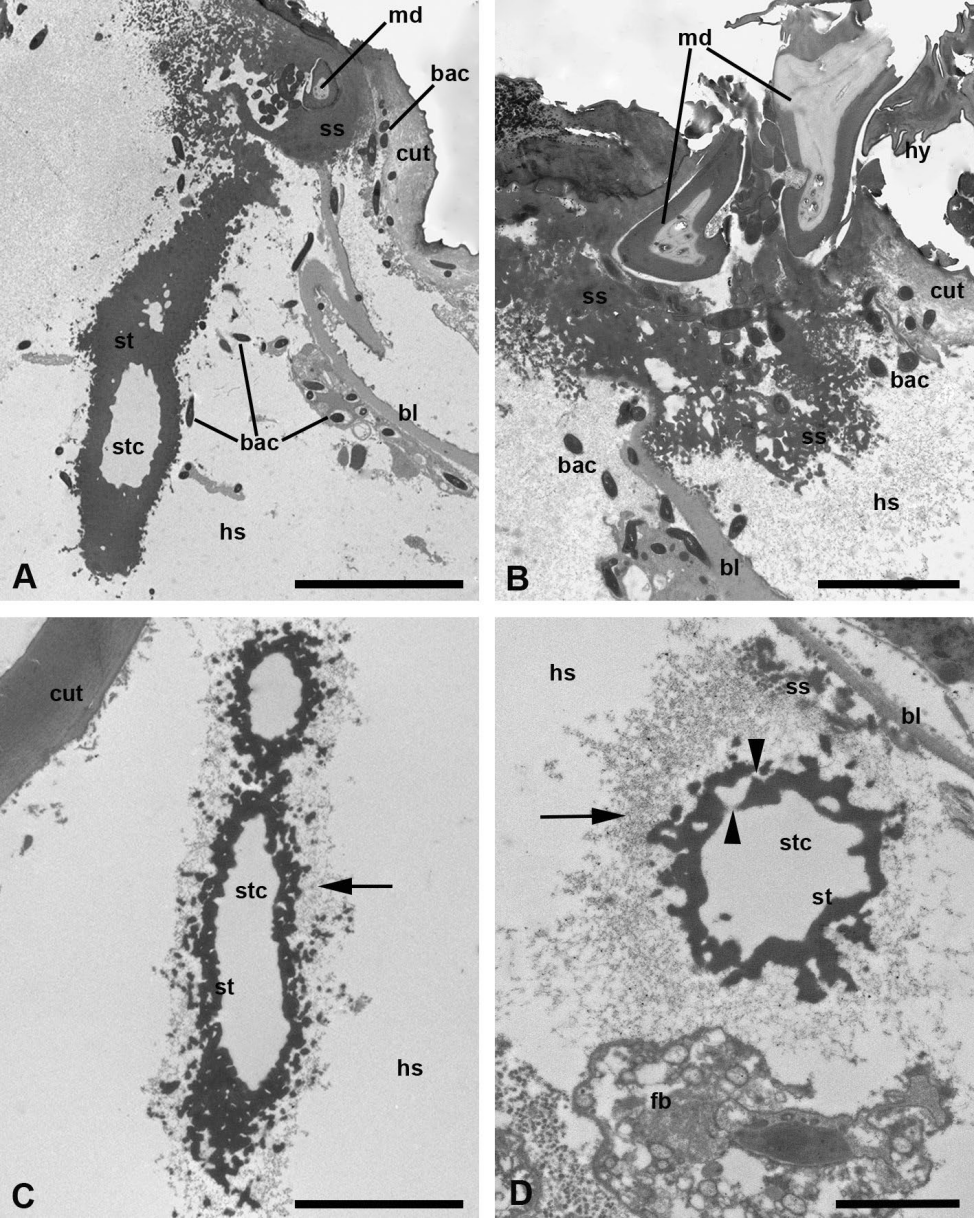
**a**–**d** Stylostome of *Allothrombium fuliginosum* larvae. TEM micrographs. **a** Main stylostome branch (main trunk) extending from the host cuticle into the host body volume. Note bacteria invading the affected area. Scale bar, 10 µm. **b** Cheliceral movable digits and the proximal stylostome portion invaded with bacteria. Scale bar, 5 µm. **c** Oblique-longitudinal section through the lateral stylostome branch under the host cuticle. Note a fine-dispersed material surrounding stylostome branch (*arrow*). Scale bar, 5 µm. **d** Cross section through the secondary or tertiary stylostome branch situated within the host tissue. Note the fine-dispersed material around the stylostome (*arrow*) and transparent canals in the stylostome walls connecting the central canal and the surrounding haemocoelic space (*arrowheads*). Scale bar, 2 µm. *bac* bacteria, *bl* basal lamina, *cut* cuticle, *fb* fat body, *hs* haemocoelic space, *md* movable digit, *ss* stylostome substance, *st* stylostome, *stc* stylostome canal

Semi-thin sections and especially TEM showed that the cheliceral movable digits of feeding larvae cut the host cuticle through (Figs. 4a, b, 5a–d). The tips of cheliceral claws are strongly moved apart so that each blade lies nearly parallel to the host cuticle on the inside looking in opposite direction (Fig. 5a). At the same time, the stylostome substance covers the movable digits (Fig. 5b, c) and may be slightly extruded to the surface of the integument. As a result, the cheliceral blades appear to be tightly enveloped by the stylostome material. A relatively wide space immediately between and underneath the movable digits is free of any contents and looks optically empty (Fig. 5a–c). Just beneath the digits, this space turns into the main stylostome canal (Figs. 4d, 5b, d). The stylostome substance, enveloping the cheliceral blades comes into the stylostome walls without any changes. The configuration of the most proximal portion of the stylostome is irregular that implies the possibility of the ramification of the canal immediately from its base (Fig. 5c). This ‘process’ may be lesser or greater expressed, so that the large irregular mass of the individual stylostome may be observed beneath the cuticle showing proximal portions of the branches extending in different directions (Fig. 5d). Sometimes, a relatively large area just beneath the cuticle in the penetration zone appears to be occupied with a dispersed stylostome material in the form of quite numerous electron-dense drops, particles, and globules (Fig. 6a, b).

The host tissues demonstrate extremely large lacunas of the haemocoelic space with a badly preserved epidermis (Fig. 4a–c), which is affected by certain components of the parasite saliva secretion. In the penetration area, the thick epidermal basal lamina of low electron density comes into contact with the stylostome substance (Figs. 5b, 6b). By contrast, immediately away from the point of penetration, the basal lamina drifts radially far apart from the cuticle. The stylostome branches may spread between the cuticle and the basal lamina or beneath the basal lamina, within the body haemocoelic space (Fig. 6a, d). Importantly, that ‘drops’ of the stylostome substance (see below) may penetrate the basal lamina, which indicates the permeability of the latter for the stylostome material (Fig. 5b).

The stylostome as such is a long branched tube-like structure spreading far away from the point of penetration and deep into the body cavity (Figs. 4a, c, 6a). The stylostome walls are without any layers or longitudinal and transverse stratifications (Fig. 6a). The walls confine the stylostome canal, which is always optically empty (Figs. 4d, 6c, d). They are built up of an electron-dense substance; the external surface is highly irregular showing ‘protuberances’ and separate drops and globules, whereas the internal one, bordering the canal, is much smoother (Fig. 6a, c). At the same time, the internal surface of the stylostome walls, especially at their bases, reveals indentations obviously manifesting the initiations of the lateral, secondary, and tertiary branches. The stylostome walls may demonstrate empty vesicles, vacuoles, and cavities of different shape and size (Fig. 5d). Through the course of the stylostome branches, their diameter progressively decreases (see above), and the walls become much more irregular from both sides even showing transparent canals and cavities (Fig. 6d). Thus, to the distal end, the stylostome canals lose their integrity, and there are immediate connections between the stylostome lumen and the surrounding hemacoelic space. As a rule, a very fine-dispersed material, nearly imperceptible, accompanies the stylostome branches (Fig. 6c, d). The stylostome canal does not reveal local dilations throughout its course that could indicate the pumping of nutrients or saliva flows. Although the walls of the most distal parts of the stylostome tubes show pores and cavities, the actual termination cannot be ascertained with certainty.

In one case, a particular bacterial contamination was observed in the area of the host tissue around a stylostome (Figs. 4d, 6a, b). No doubt, these bacteria originated from the parasitizing larva, because (i) they were seen immediately in the zone between the bases of the cheliceral blades (see above) (Fig. 6b), i.e., they were introduced by the larva, and (ii) they invaded only the space in the vicinity of the given stylostome. These bacteria, apparently long and dividing, could be seen within the stylostome substance, especially, in its proximal portion revealing the particular viscosity, sufficient for the bacterial movement. These bacteria may be also identified within the host cells and even cuticle (Fig. 6a, b).

## Discussion

### Intra-generic consistency of the stylostome structure

The structure of the stylostome produced by *A. fuliginosum* larvae is similar to one observed in *A. recki*, which corroborates the morphological consistency of the feeding canals at the intra-generic level. According to the rough description provided by Feider and Agekian (1967), the stylostome of *A. recki* consists of 10–12 canals, the longest of which is 250 µm long, the maximum diameter of the stylostome is 6 µm, whereas the diameter of the axial canal is 1.5 µm. In the stylostome formed by *A. fuliginosum*, we could state the presence of 2–12 canals (main and secondary branches), depending on the age of the structure. The maximum diameter of the stylostome as well as the diameter of the canal lumen varied to a higher extent, which may be attributed to the higher sample size considered in the present study. Due to the overall similarity of the stylostomes, hitherto described for two members of *Allothrombium*, but also due to several factors that may contribute to the variation of the feeding canals (with special reference to the age and host impact) there is no background to consider the stylostome as species-specific. The latter seems to reflect the opposite mode to one reported by Mohamed and Hogg (2004) for water mites, in which the stylostomes can vary even between the closely related taxa (at intra-generic level).

### Inter-generic differences in morphology of the stylostomes

The clear differences observed at the inter-generic level, pertain to the stylostomes of two trombidiid genera, i.e., *Allothrombium* and *Trombidium*. Flögel (1876) was the first to provide a drawing of the feeding canals produced by a larva which parasitized *Erigone dentipalpis* (Wider) (Araneae). Despite the lack of systematic affiliation of the parasite, the morphological characteristics but also the data on mite ecology provided by Flögel (1876), indicate that the author dealt with *T. brevimanum*. A strong argument for supporting this hypothesis was the identity of the host, due to the confirmed associations between *T. brevimanum* and spiders (Wohltmann 1999; Judson and Mąkol 2011; Tomić et al. 2015; Felska et al. 2018).

Both, in the case of *Trombidium* spp. (Wohltmann 2000; Judson and Mąkol 2011; Shatrov and Felska 2017), and of *Allothrombium* spp. the stylostomes are branched; however, the branches are distinctly less numerous in *Allothrombium* and do not form a ‘root-like’ structure. Neither in *A. recki* nor in *A. fuliginosum* the terminations of canals are expanded into knobs (cluster of closed bulbs), the character typical for stylostomes organization in *Trombidium* (Wharton 1954; Mohamed and Hogg 2004). The stylostome branches of *T. holosericeum*, which become ramified also immediately from the stylostome base, are much shorter than these of *A. fuliginosum* and do not spread deep into the body cavity. The feeding canals attenuate in their distal parts in the stylostome formed by *A. fuliginosum*, thus an ultimate answer to the question of blind versus open-ended terminations of the feeding system cannot be given with the use of light microscopy. The fact that *A. fuliginosum* larvae detached from the host immediately after being placed in the preservative, indicates a relatively weak connection with the host compared to the one observed by Shatrov and Felska (2017) in *T. holosericeum*.

The TEM showed that the stylostome ultrastructure of *A. fuliginosum* larvae differs from that found in *T. holosericeum* feeding on larvae of *Stenodemini* sp. (Heteroptera, Miridae) (Shatrov and Felska 2017). In the latter trombidiid species, the walls of stylostome branches are wider and more solid without obvious lucent vesicles and cavities, and have much smoother outlines from both outside and inside. Moreover, through the entire length, an electron-dense granular material sometimes hardly distinguishable from the proper stylostome substance accompanies the stylostome branches. When the stylostome branch is ending distally, new canals start through this granular substance. The stylostome canal in *T. holosericeum* is filled with a fine-granular material of moderate electron density, in particular, in the proximal stylostome portion (Shatrov and Felska 2017), which is not the case in *A. fuliginosum*.

### Differences in stylostome structure at the inter-family level

The observed stylostomes in larvae of trombidiid mites differ significantly from these of trombiculid mites parasitizing vertebrates (Shatrov 2000, 2009; Shatrov and Stekolnikov 2011; Shatrov et al. 2014; Shatrov and Mirolubov 2015). In trombiculid larvae, stylostome forcedly going through the thick host epidermal layer is always straight, even if it reaches the much looser organized connective tissue layer. Its distal end is always opened. The most proximal stylostome portion is formed of the so-called eosinophil cone – the first portion of the parasite saliva discharged on the host epidermal surface, to which the cheliceral blades adhere. In contrast to trombidiid larvae, the stylostome substance in trombiculids is electron clear but also without any layers in TEM preparations (Shatrov and Felska 2017). This indicates differences in the chemical composition of stylostomes between the members of both families.

Generally, the principles of stylostome formation, especially on the initial stages, are found to be identical, thus not dependent on the systematic affiliation of the larvae. The cheliceral blades cut through the cuticle, and just after that, the larva ejects the first saliva portion that seals the wound and adheres the chelicerae to the host cuticle.

Formation of the stylostome probably takes place during the first hours after getting in contact with the host. The *Arrenurus* stylostome (consisting of a single-blind tube) begins to form within 10 min after attachment and attains its final shape within 1 day (Åbro 1979; Smith 1988), which is considerably faster compared to species forming multibranched stylostomes (Davids 1973 for *Hydrachna*). In the case of *A. fuliginosum*, the initial stylostome formation was visible in the host's tissues 10 h after attachment.

### Morphology of the feeding apparatus in mites

The mouth apparatus in *A. fuliginosum* larvae appears to be more generalized externally than that in *T. holosericeum* (Shatrov and Felska 2017) and some other trombidiid species (Shatrov 2011), because the apical hypostome portion does not form a permanent sucker. By this character, the mouth apparatus is rather similar to trombiculid larvae, in which the labile apical hypostome lips turn back during feeding forming only a temporary sucker tightly pressing itself against the host epidermis and favoring the work of the pharyngeal pump (Shatrov et al. 2016). Interestingly, in both trombiculid larvae and larvae of *A. fuliginosum*, this temporary sucker is devoid of accompanying setae. In contrast to the majority of trombiculid larvae provided with a tri-furcate palpal claw, the larvae of *A. fuliginosum* possess a bi-furcate claw; in either case, the claw is not involved in piercing the host cuticle (Shatrov et al. 2016).

Comparison with the highly diverse group of water mites (Hydrachnidia) shows that generally the mouth apparatus of *A. fuliginosum* larvae is rather simpler organized than that of water mite larvae (Vainstein 1980). It is noteworthy at the same time that the palp tarsus in *A. fuliginosum* is armed with the spoon-like solenidion oriented medially and similar to one observed in water mite larvae *Piona carnea* (Koch) (Shatrov 2012).

### Effect of the host on stylostome structure

Little research has been done on the effect of the host species or its size on the morphology of the stylostome. Most topic-related data concern water mites. Lanciani and Smith (1989) compared the stylostomes produced by two *Arrenurus* species (*Arrenurus novimarshallae* Wilson and *Arrenurus pseudotenuicollis* Wilson), both of which parasitized two mosquito species. The overall similarity of the stylostomes formed by conspecific larvae in different host species pointed to the species-specific nature of the feeding canals. Although the stylostomes produced by *Ar. novimarshallae* remained constant with respect to the general shape, they were found significantly smaller in *Anopheles quadrimaculatus* Say due to the restrictions imposed by the host's immune response (Lanciani and Smith 1989). Our comparison of stylostomes produced in the tissues of three host species also revealed the high consistency in the shape of these structures among hosts. However, the total length of the stylostome differed significantly among the examined host species. The differences did not positively correlate to the hosts’ body size as the longest stylostomes were present in *Ac. pisum*, an intermediate species between the larger *M. rosae* and smaller *E. abietinum*. This is probably due to the preference of *A. fuliginosum* for *Ac. pisum*, but further research is needed to test this hypothesis. Preference of *Allothrombium* larvae for particular host species has hitherto been examined by Zhang (1996) and Hosseini et al. (2002) only in relation to *Allothrombium pulvinum* Ewing.
